# Does the Floral Nectary in *Dracocephalum moldavica* L. Produce Nectar and Essential Oil? Structure and Histochemistry of the Nectary

**DOI:** 10.3390/biology11111650

**Published:** 2022-11-11

**Authors:** Agata Konarska, Elżbieta Weryszko-Chmielewska, Marta Dmitruk, Aneta Sulborska-Różycka, Krystyna Piotrowska-Weryszko

**Affiliations:** Department of Botany and Plant Physiology, University of Life Sciences, Akademicka 15, 20-950 Lublin, Poland

**Keywords:** Lamiaceae, nectaries, Moldavian balm, micromorphology, anatomy, ultrastructure, secondary metabolites

## Abstract

**Simple Summary:**

*Dracocephalum moldavica* is an aromatic plant originating from Asia and grown for medicinal, cosmetic, seasoning, beekeeping, and decorative purposes. All types of trichomes present in the aboveground parts of the plant produce essential oil. The floral nectaries of this species have special properties, as they produce not only nectar but also essential oil, which we have described in the present study. The micromorphological studies have shown that nectar release is mediated by nectarostomata, and the histochemical tests have revealed that the essential oil is produced by trichomes and the pavement cells of the nectary epidermis. The secretion of aromatic nectar has great ecological importance in the plant–insect relationship and explains the great interest of bees in the flowers of this species. Our study provides the first description of the production of nectar and essential oil by the nectaries in the family Lamiaceae.

**Abstract:**

*Dracocephalum moldavica* is an aromatic plant with a lemon scent and versatile use. Its flowers produce large amounts of nectar, which is collected by bees and bumblebees. The aim of the study was to investigate the structure of the floral nectary in this melliferous plant, which has not been analysed to date. The analyses were carried out with the use of light, fluorescence, scanning electron, and transmission electron microscopy, as well as histochemical techniques. The four-lobed nectary with a diameter of 0.9–1.2 mm and a maximum height of 1.2 mm is located at the ovary base; one of its lobes is larger than the others and bears 20–30 nectarostomata and 8–9 glandular trichomes. The histochemical assays revealed the presence of essential oil and phenolic compounds in the nectary tissues and in glandular trichomes. The nectary tissues are supplied by xylem- and phloem-containing vascular bundles. The nectariferous parenchyma cells have numerous mitochondria, plastids, ribosomes, dictyosomes, ER profiles, vesicles, thin cell walls, and plasmodesmata. Starch grains are present only in the tissues of nectaries in floral buds. The study showed high metabolic activity of *D. moldavica* nectary glands, i.e., production of not only nectar but also essential oil, which may increase the attractiveness of the flowers to pollinators, inhibit the growth of fungal and bacterial pathogens, and limit pest foraging.

## 1. Introduction

Many species of the family Lamiaceae are aromatic plants producing essential oils, which are secreted by glandular trichomes located on the leaves, stems, and flowers [1,2]. *Dracocephalum moldavica* L. has a strong lemon scent associated with the substantial amounts of citral contained in its oil [3,4]. As shown in our previous study, the essential oil in the leaves of this species is contained not only in various types of glandular and non-glandular trichomes but also in pavement epidermal cells [4].

With its phytochemical properties, *D. moldavica* has many applications. It is used as a medicinal, cosmetic, seasoning, and ornamental plant [5,6]. It is also one of the highly valued melliferous plants, as it produces large amounts of nectar with high sugar content [7,8,9]. Tea blends/infusions from *D. moldavica* aerial parts are used for medical purposes. The plant is used in the production of perfumes, soaps, and detergents in the cosmetic industry and for the aromatisation of jams, candies, syrups, canned fish, and alcohol in the food industry [10].

*Dracocephalum moldavica* produces purple-blue or white zygomorphic flowers. The approximately 20 mm long corolla has a long tube where the nectar accumulates and sometimes fills the tube completely. The nectar is most often used by bees and bumblebees [8]. It is the main food attractant for insect visitors and a source of energy, sugars, and other nutrients, e.g., amino acids and minerals, as well as water [11].

The nectary gland in Lamiaceae flowers forms a ring at the base of the superior ovary [12]. It differs in different taxa in size, symmetry, shape, and number of protrusions [13,14,15]. The ring may be unlobed as in *Prasium majus* [14] and *Origanum vulgare* [15]. However, it most often has four lobes of various sizes, i.e., there may be one lobe longer than the others in *Salvia farinacea* [16] and *Melissa officinalis* [17], three longer lobes and a shorter one in *Ocimum basilicum* [18], or four equally sized lobes in *Thymus capitatus* [14] and *Mentha spicata* [15]. Nectar in Lamiaceae is released onto the nectary surface through nectarostomata, which are most often located on the abaxial part of the nectary [13,14,16,17,19,20].

Nectaries of some Lamiaceae species bear glandular trichomes. They were observed in *Scutellaria baicalensis* [21], *Scutellaria pinnatifida* [22], *Marrubium parviflorum* [23], and *Salvia farinacea* [16].

The aim of the present study was to analyse the micromorphology, anatomy, and ultrastructure of the *D. moldavica* floral nectary, which produces large amounts of nectar and is thus an attractive species for bees. We also applied several histochemical assays to achieve a more complete characterisation of the nectary. We especially focused on the structure of the nectary epidermis. An additional aim of our study was to check whether the nectary and nectar guides present on corolla petals produce volatile substances, as the attractiveness of flowers to insect pollinators is associated with the presence of not only food attractants but also visual and/or aromatic substances that have an impact the interactions between the pollinator and the plant. Noteworthy, the ultrastructure of the nectary glands in Lamiaceae species has been poorly investigated to date, and the present study provides some new aspects in this field. The localisation and structure of nectaries may play a role in taxonomy and evolution and may contribute to the development of research on the ecology of the species.

## 2. Material and Methods

### 2.1. Plant Material

*Dracocephalum moldavica* L. flowers were subjected to morphological and anatomical analyses. The plants were collected in 2021–2022 from the Botanical Garden of Maria Curie-Skłodowska University in Lublin (51°15′44′′ N, 22°30′48′′ E). The voucher specimens were deposited in the Herbarium of the Department of Botany and Plant Physiology (University of Life Sciences in Lublin). Additionally, the correctness of the identification of the taxon was confirmed by taxonomy specialist Professor Bożena Denisow.

**Figure 1:**
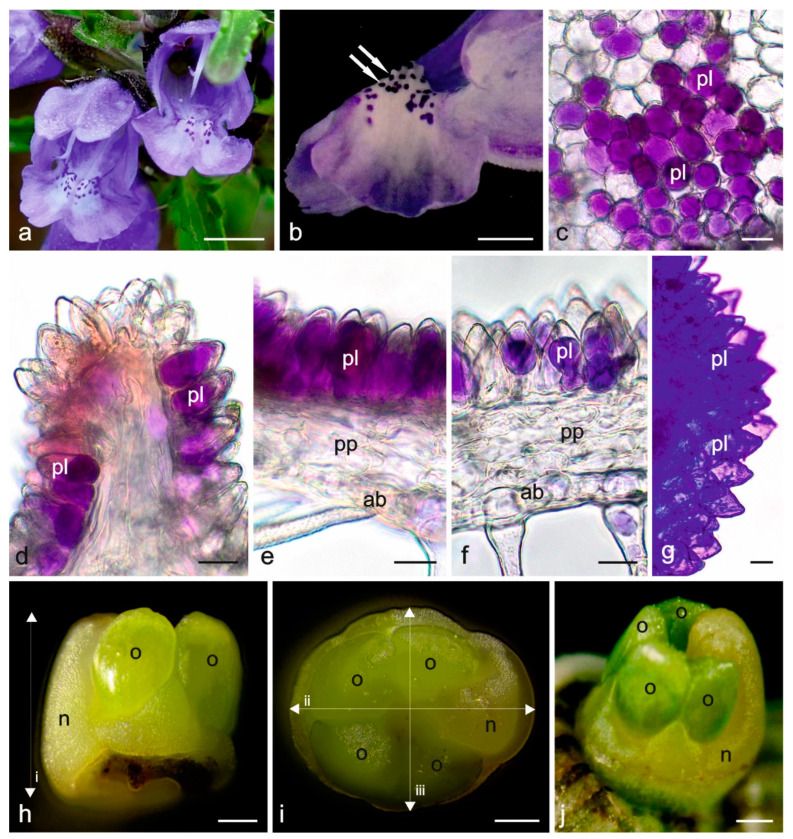
*Dracocephalum moldavica* flowers and their fragments. (**a**) Part of the inflorescence; (**b**) Flower guides (white double arrow) on the lower lip of the flower; (**c**) Epidermal cells (papillae) forming flower guides—top view, fresh flower; (**d**) Some flower guides located on corolla protrusions—cross-section, fresh flower; (**e**) Cross-section of the lower lip with papillae forming a flower guide—fresh flower; (**f**) Cross-section of the corolla at the flower guide margins, anthocyanin-stained vacuoles in epidermal cells—fresh flower; (**g**) Papillae on the surface of the lower lip stained with Nile blue; (**h**–**j**) Four-lobed ovaries and annular nectaries collected from fresh flowers: (**h**,**i**)—closed bud stage, (**j**)—anthesis stage; pl—papillae; pp—petal parenchyma; ab—abaxial epidermis; n—nectary; o—ovary; i—height of the longer nectary lobe; ii—longer diameter of the nectary; iii—shorter diameter of the nectary. Scale bars: 500 µm (**a**), 200 µm (**b**,**h**–**j**), 20 µm (**c**–**g**).

The flowers (*n* = 10) were picked from different plants in two development stages each year: (i) the bud stage and (ii) full flowering on the 1st day of anthesis. They were collected from thyrses growing in the third inflorescence whorl from the bottom. The height of the highest part of the nectary and its longer and shorter diameters were measured (Figure 1h,i). The micromorphology of the lower lip and the structure of floral nectaries were analysed using stereoscopic (SM), light (LM), and fluorescence (FM) microscopy, as well as scanning electron microscopy (SEM) and transmission electron microscopy (TEM).

### 2.2. Stereoscopic Microscopy (SM)

The initial studies of the structure of the flowers, the location of the nectaries as well as measurements of the nectary size were carried out in fresh material with the use of an Olympus SZX2-ILLT stereomicroscope (Olympus, Tokyo, Japan). The photographs were taken with the use of the Olympus cellSens Standard software ver. 2.1.17342.0 (Olympus, Tokyo, Japan).

### 2.3. Scanning Electron Microscopy (SEM)

The nectaries (*n* = 10) for the analyses were collected from different flowers and plants. The samples were fixed in 4% glutaraldehyde in 0.1 M sodium phosphate buffer (pH 7.0) for 12 h at room temperature. Next, they were washed in the same buffer four times at 20 min intervals and dehydrated in increasing concentrations of an acetone series (30, 50, 70, 90, 95%). The dehydrated samples were critical-point dried in liquid CO_2_ using Bal-Tec CPD 030 (Bal-Tec, Balzers, Liechtenstein). The plant material prepared in this way was mounted onto stubs and gold sputter coated (thickness approx. 10 μm) using a Emitech SC 7640 sputter coater (Polaron, Newhaven, East Sussex, UK). The specimens were analysed under a TESCAN/VEGA LMU (Tescan, Brno, Czech Republic) scanning electron microscope at an accelerating voltage of 30 kV.

### 2.4. Light Microscopy (LM)

*Dracocephalum moldavica* flowers were collected for histological and cytological studies. The material was sampled randomly from 20 plants. Hand-made cross-sections of the fresh lower lip and cross- and longitudinal sections of the nectary were prepared.

To prepare semi-thin sections (0.7–0.9 μm), nectaries were collected from different flowers growing on different plants and fixed in 4% glutaraldehyde in phosphate buffer (pH 7.2; 0.1 M) for 12 h at 4 °C. In the following step, the specimens were washed three times in phosphate buffer. After dehydration in an ethanol series, they were embedded in LR white resin (LR white acrylic resin, medium grade, Sigma-Aldrich, St. Louis, MO, USA) and cut longitudinally with a glass knife of the Reichert Ultracut S ultramicrotome (Reichert-Yung, Vienna, Austria). Semi-thin sections were stained with a 1% aqueous methylene blue-azure II solution [24].

All slides were analysed with the use of a Nikon Eclipse 400 light microscope (Nikon, Tokyo, Japan) with a digital camera Coolpix 4500 (Nikon) and an Olympus CX 23 light microscope (Olympus, Tokyo, Japan) with an Olympus EP50 digital camera (Olympus) and EP view software.

### 2.5. Histochemistry and Fluorescence Assays

Manual longitudinal and cross-sections of fresh fragments of nectaries with ovaries from flowers in the full bloom stage were made using a razor blade. The following histochemical assays were carried out to detect the metabolites present in the nectary cells: Sudan IV to detect total lipids [25,26], Nile Blue to stain acidic and neutral (essential oil) lipids [27,28], and toluidine blue O to stain phenolic compounds [24], and Lugol’s solution to detect neutral polysaccharides (starch) [29] in the nectary cells and in the glandular trichomes present on the nectary surface. Additionally, the cross-sections of fresh fragments of the lower lip with nectar guides were treated with Nile blue to stain essential oil. The stained sections (5 repetitions for each method) were examined and imaged under a Nikon SE 102 light microscope (Nikon). Standard control procedures suggested by the aforementioned authors were applied simultaneously. Moreover, the sections of nectaries were embedded in water with glycerol (1:1) and examined by means of fluorescence microscopy to determine the location of phenolic acids [30,31] and in aluminium chloride fluochrome to detect the presence of flavonoids [32]. Light blue autofluorescence of phenolic acids excited with UV radiation and the light-yellow fluorescence of flavonoids was observed by means of a Nikon Eclipse 90i microscope (Tokyo, Japan) coupled with a digital camera (Nikon Fi1) (Nikon) and NIS-Elements Br 2 using a Cy5 filter (excitation wavelength 590–650 nm) and a barrier filter (wavelength 663–738 nm).

### 2.6. Transmission Electron Microscopy (TEM)

Nectaries were collected from fresh flowers (*n* = 5) in the full bloom stage and fixed as described above for the semi-thin sections. Next, the samples were post-fixed in a 1% osmium tetraoxide solution for 1.5 h at 0 °C and washed three times in distilled water. The plant specimens prepared in this way were dehydrated in a graded ethanol series and embedded in LR white resin (as above). After polymerisation at 60 °C, the embedded material was cut into ultrathin sections (60 to 90 nm) using a Reichert Ultracut S ultramicrotome (C. Reichert, Vienna, Austria) and a glass knife. In the next step, the specimens were stained with 0.5% uranyl acetate and post-stained with 0.5% lead citrate [33]. The material was analysed with the use of a BS-500 Tesla (Tesla, Brno, Czech Republic) transmission electron microscope.

## 3. Results

### 3.1. Visual and Aromatic Floral Attractants

The blue (Figure 1a) or purple *D. moldavica* corolla has a distinctive colouration of the lower lip (Figure 1b). It has clusters of dark purple spots on a light background, acting as flower guides. These spots are clearly visible from above, thus attracting insects approaching the flowers. They are located at the corolla tube inlet, indicating the entrance to the nectar-containing interior of the flower (Figure 1a,b).

The surface of the lower lip is slightly undulated. The cross-section of the epidermis within the spots on the petal shows the presence of long papillae (35–41 µm), which are mainly occupied by a centrally located vacuole filled with purple cell sap (Figure 1c–f), and they produce essential oil (reaction with Nile blue) (Figure 1g). The papillae within the spots constitute nearly half of the thickness of the petal. Some spots are located on petal protrusions (Figure 1d). Papillae are also present on the entire adaxial surface of the upper and lower lips, but they are shorter and have fewer pigmented vacuoles than those in the spots. Papillae surrounding the spots are colourless (Figure 1c).

The abaxial surfaces of both lips bear flat epidermal cells and various types of trichomes: peltate and capitate glandular trichomes as well as non-glandular trichomes. The peltate trichomes are composed of one basal cell, a unicellular stalk, and 8–12 secretory cells forming the head. The capitate trichomes are formed by one basal cell, a 2–3-celled uniseriate stalk, and a unicellular glandular head (not shown).

### 3.2. Micromorphology of Nectary

**Figure 2:**
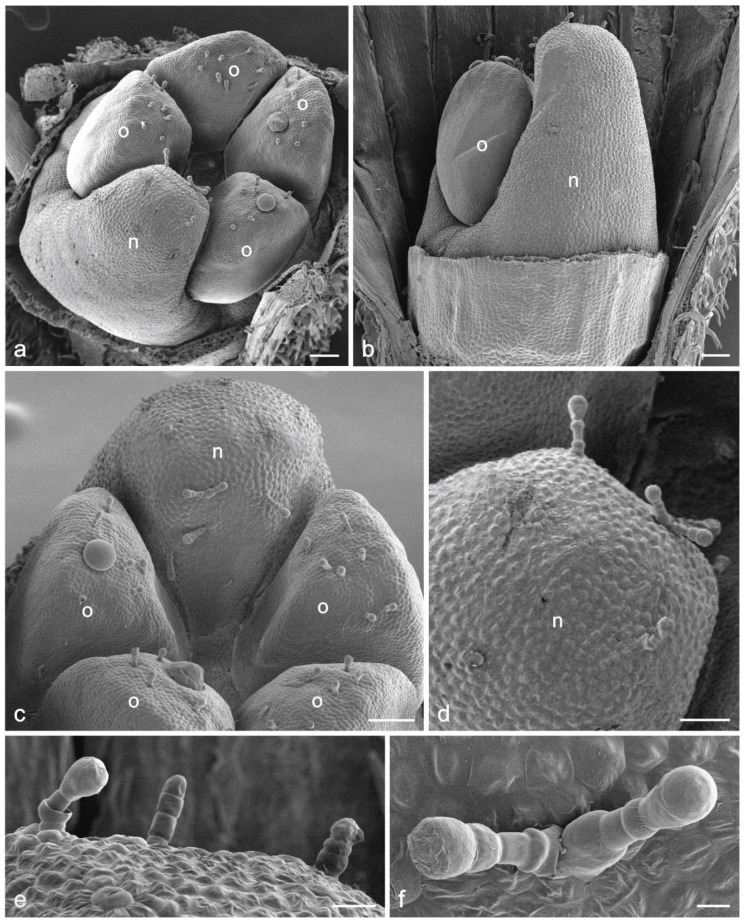
Scanning electron microscopy (SEM) micrographs of *Dracocephalum moldavica* floral nectary and ovary. (**a**) Ovary with the nectary in top view; (**b**) Abaxial surface of the longer nectary lobe and ovary part; (**c**) Different glandular trichomes on the top of the longer nectary lobe and on the ovary parts; (**d**) Upper part of the longer nectary lobe with glandular trichomes; (**e**,**f**) Trichomes on the nectary surface; o—ovary, n—nectary. Scale bars: 100 µm (**a**–**c**), 50 µm (**d**), 20 µm (**e**), 10 µm (**f**).

The nectary in *D. moldavica* flowers is located at the base of the ovary (Figure 1h–j). It is a four-lobed asymmetrical disc with one longer lobe facing the lower flower lip. This lobe is higher than the ovary (Figure 1j and Figure 2a–c).

During the flower development, the longer and shorter diameters of the nectary increased by over 20%, whereas the height of the longer lobe increased by 8% (Table 1). The nectary was light yellow in the closed bud stage (Figure 1h,i) and intensely yellow in the open flower stage (Figure 1j), which clearly distinguished it from the green ovary.

**Figure 3:**
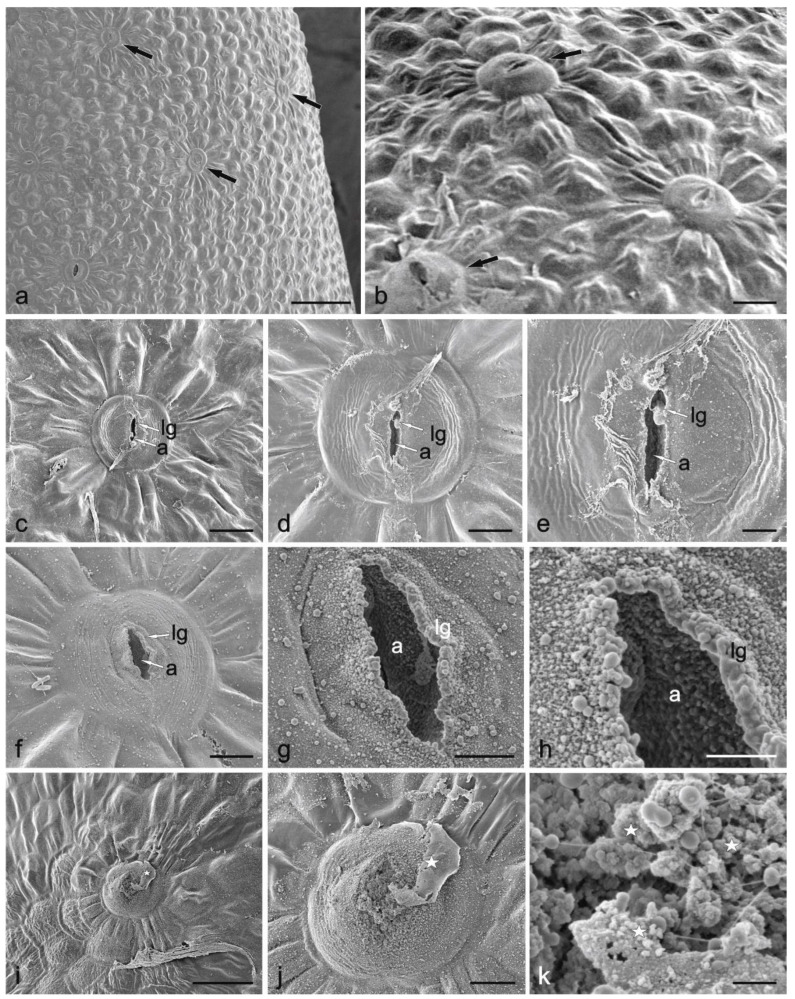
Nectarostomata on the abaxial surface of the longer lobe of the *Dracocephalum moldavica* nectary (SEM). (**a**) A part of the epidermis with nectarostomata before nectar secretion (arrows); (**b**) Nectarostomata with an open pore at the beginning of nectar secretion (arrows); (**c**–**e**) Stoma in the aperture and ledge formation stage (various magnifications); (**f**–**h**) Stoma with formed ledges during nectar secretion (various magnifications); (**i**–**k**) Stoma with an aperture occluded by nectar residues (stars); a—aperture; lg—ledge. Scale bars: 50 µm (**a**), 20 µm (**i**), 10 µm (**b**,**c**), 5 µm (**d**,**f**,**j**), 2 µm (**e**,**g**), 1 µm (**h**), 0.5 µm (**k**).

There were 8–9 glandular capitate trichomes on the large nectary lobe. The trichomes were situated on the top and the adaxial part of the lobe. The glandular trichomes were most often arranged singly and less often in pairs (Figure 2c–f). They consisted of 1 basal cell, 2–4 stalk cells, and 1 spherical or slightly elongated head cell. In turn, there were some peltate and capitate trichomes on the ovary parts (Figure 1c). There were 20–30 nectarostomata only on the longer nectary lobe (Figure 3a,b). They were visible on the top and on the abaxial surface of this lobe. These solitary structures were located at certain distances from each other (Figure 3b). The modified stomata were visible slightly below the other epidermal cells before the nectar release stage and above the other epidermal cells during the nectar release stage (Figure 3b,i,j). The stomatal complex was composed of two guard cells and 7–8 subsidiary cells (Figure 3b,c). Cuticular striation was visible on the surface of the nectarostomata (Figure 3d,e). The small amounts of nectar residues on the surface of some guard cells (Figure 3f–h) and their substantially larger amounts on other stomata and neighbouring epidermal cells (Figure 3i,j) indicate the non-synchronous functioning of the nectarostomata.

### 3.3. Anatomy of the Nectary

**Figure 4:**
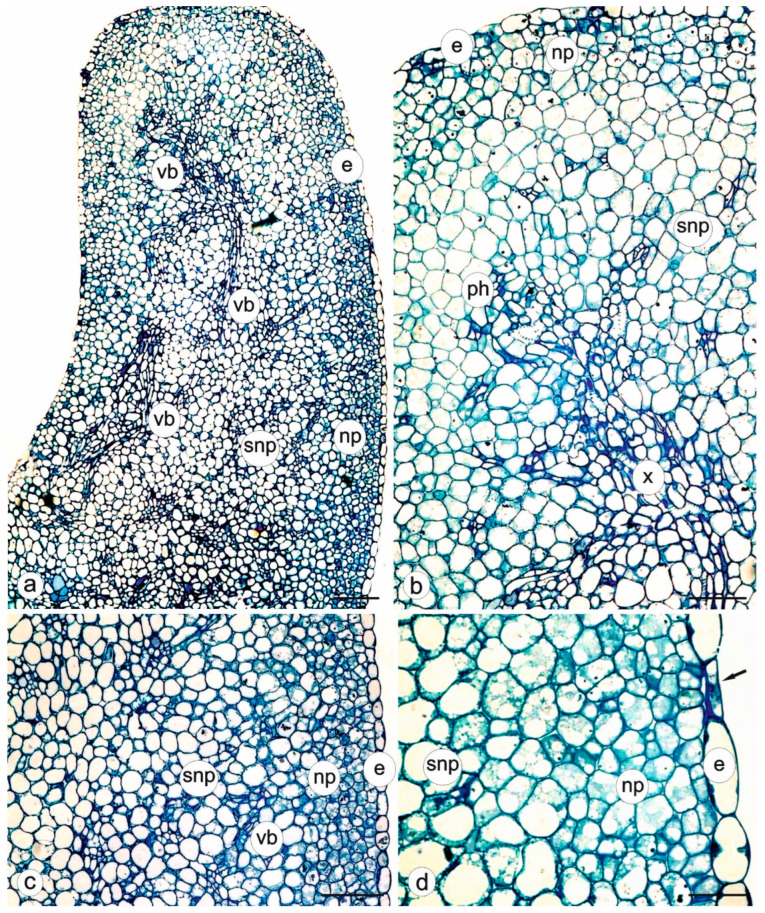
Anatomy of the floral nectary of *Dracocephalum moldavica*—light microscope (LM). (**a**) Longitudinal section of the longer nectary lobe; (**b**) Higher magnification of figure (**a**) showing branched phloem and xylem strands in subnectary parenchyma; (**c**) Fragment of the section of the lateral nectary part with visible epidermis, nectary parenchyma, and subnectary parenchyma as well as strands of vascular bundles; (**d**) Epidermis with stoma (arrow), nectariferous parenchyma, and subnectary parenchyma; e—epidermis; np—nectary parenchyma; snp—subnectary parenchyma; x—xylem; ph—phloem; vb—vascular bundle. Scale bars: 100 µm (**a**), 50 µm (**b**,**c**), 20 µm (**d**).

The longitudinal section of the nectary tissue is shown in Figure 4a,b. The nectary epidermis had thin-walled cells with a high degree of vacuolation in the anthesis stage (Figure 4c,d). The nectar-producing parenchyma constituted a sub-epidermal layer composed of 5–6 layers of small cells. They were arranged irregularly and were characterised by the presence of thin walls, a dense cytoplasm, and mostly a low vacuolisation degree (Figure 4c,d). The subnectary parenchyma occupied the central part of the nectary lobes. It was formed by larger cells than those in the nectariferous parenchyma. These cells had large vacuoles and parietal cytoplasm (Figure 4c,d). The vascular bundles had multiple branches. They were localised only in the subnectary parenchyma and comprised xylem and phloem elements (Figure 4b,c).

### 3.4. Histochemistry

**Figure 5:**
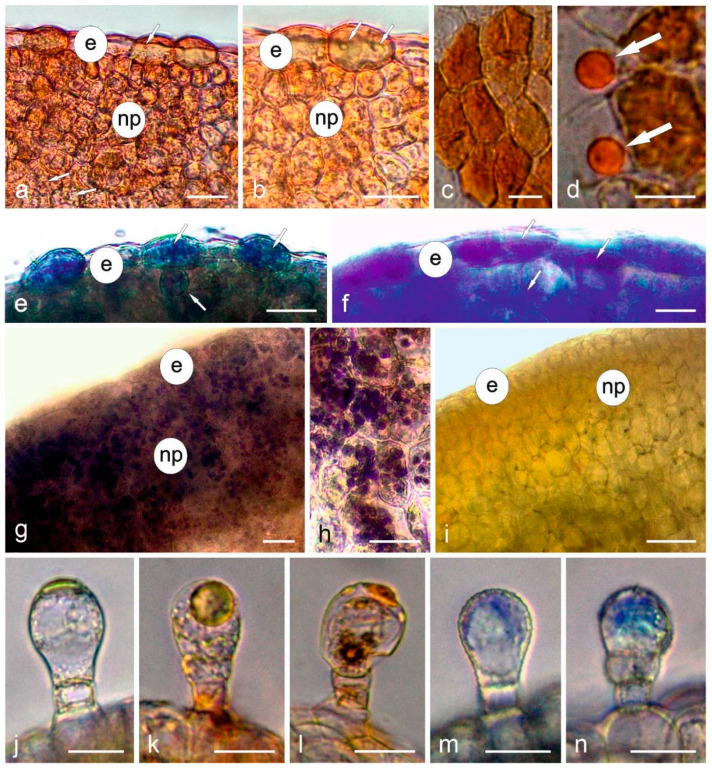
Results of histochemical tests of *Dracocephalum moldavica* nectary cells. Light microscopy (LM)—cross-sections. (**a**,**b**) Lipid compounds stained orange with Sudan IV in epidermal (white arrows) and parenchyma cells; (**c**,**d**) Lipid compounds in epidermal cells stained orange with Sudan IV—top view. Note the large lipid droplets (white arrows); (**e**) Acid lipids stained blue with Nile blue in the epidermis and parenchyma (white arrows); (**f**) Neutral lipids (essential oil) stained purple with Nile blue present in epidermal and parenchyma cells (white arrows); (**g**,**h**) Neutral polysaccharides (starch grains) stained purple with Lugol’s solution in nectary parenchyma cells in the swollen bud stage; (**i**) Starch-free nectary cells treated with Lugol’s solution in a freshly opened nectar-secreting flower; (**j**–**n**) Glandular trichomes on the nectary surface; (**j**)—control, in water; (**k**,**l**) Glandular trichomes treated with Sudan IV with visible orange content; (**m**,**n**) Phenolic compounds stained with toluidine blue in trichome heads; e—epidermis; np—nectary parenchyma. Scale bars: 50 µm (**i**), 20 µm (**a**,**b**,**e**,**g**,**h**,**j**–**n**), 10 µm (**c**,**d**,**f**).

**Figure 6:**
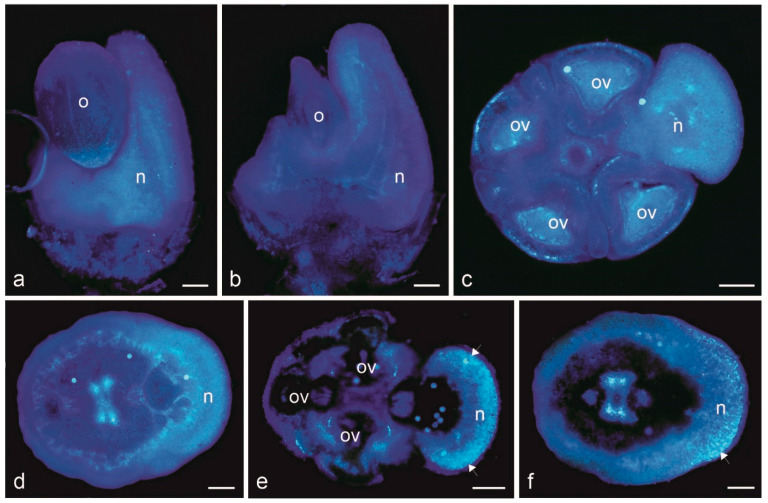
Results of fluorescence assays of *Dracocephalum moldavica* nectary cells. (**a**,**b**)—a surface side view; (**c**–**f**) cross–sections. (**a**–**d**) Intense light blue autofluorescence of phenolic acids visible in the nectary cells; (**e**,**f**) Light yellow fluorescence of flavonoids in the presence of aluminium chloride fluochrome visible in the nectary epidermis and parenchyma (arrows); n—nectary; o—ovary; ov—ovules. Scale bars: 200 µm (**a**–**f**).

The histochemical and fluorescence assays revealed the presence of lipid and phenolic compounds in the cells and trichomes of the Moldavian balm nectary (Figure 5 and Figure 6).

Both total lipids stained orange with Sudan IV (Figure 5a–d) and acid lipids stained blue with Nile blue (Figure 5e) were detected in the nectary epidermal and parenchyma cells, while neutral lipids (essentials oil) stained purple with Nile blue were mainly located in the epidermal cells (Figure 5f). The lipid-accumulating epidermal cells were larger than the neighbouring cells, and some of them contained large lipid droplets (Figure 5d). Lipids were also present in the content of the trichome head cells (Figure 5k,l). Numerous Lugol’s solution-stained starch grains were visible in the nectary parenchyma in the swollen bud stage (Figure 5g,h), whereas no starch grains were detected in the freshly opened flowers releasing nectar (Figure 5i). The nectary tissues were also the site of accumulation of phenolic acids, whose intense light blue autofluorescence was observed in the nectary parenchyma (Figure 6a–d). In turn, in the presence of the aluminium chloride fluochrome, the flavonoids located in the epidermis and parenchyma of the *D. moldavica* nectary emitted intense yellow fluorescence (Figure 6e,f). Phenolic compounds were also detected in the nectary glandular trichomes after the application of toluidine blue O (Figure 5m,n).

### 3.5. Ultrastructure of the Nectary Cells

**Figure 7:**
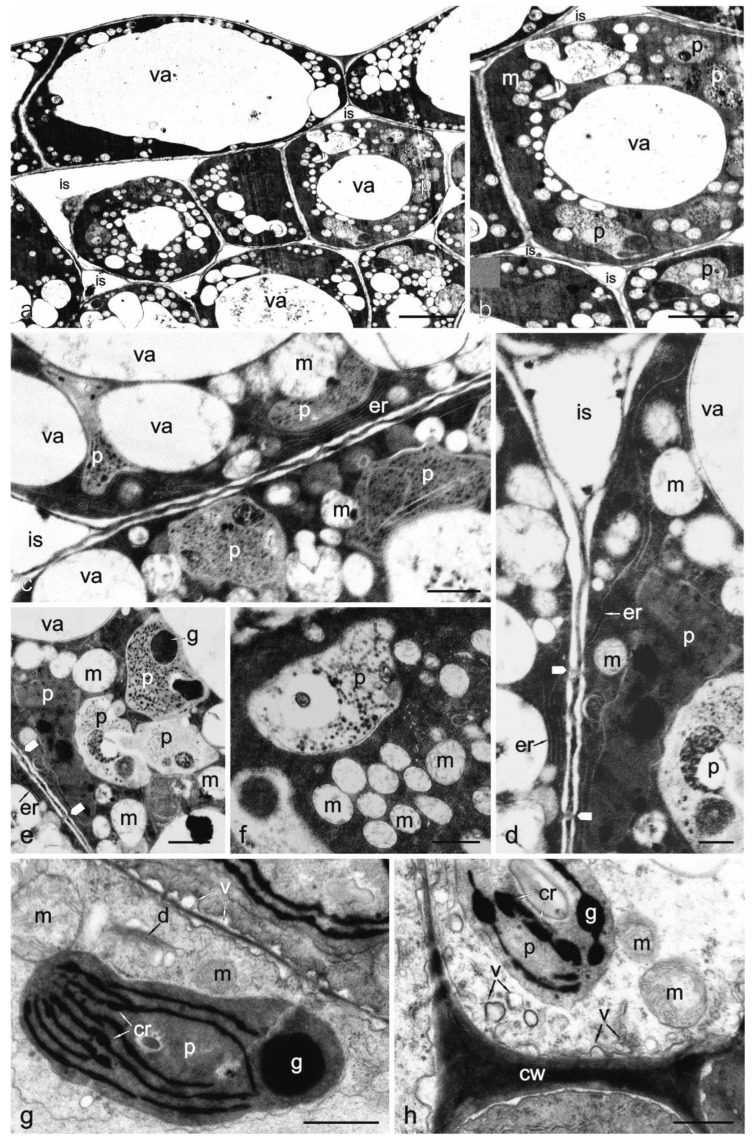
Ultrastructure of the floral nectary *Dracocephalum moldavica* (TEM). (**a**) Vacuolated epidermal and parenchyma cells of nectary with electron-dense cytoplasm, numerous mitochondria with a transparent matrix, and intercellular spaces of different sizes. (**b**) Nectary parenchyma cells with dense cytoplasm, many plastids, mitochondria, and vacuoles; (**c**) Parts of two nectary parenchyma cells with pleomorphic plastids with small plastoglobuli, mitochondria, and vacuoles; (**d**) Plastids with plastoglobuli, mitochondria, vacuoles, smooth ER, and plasmodesmata (white arrowheads) in the wall of nectariferous cells; (**e**) Cluster of plastids with large plastoglobuli, some of them connected, mitochondria and plasmodesmata (white arrowheads); (**f**) Numerous mitochondria with distinct cristae and plastids in the dense cytoplasm; (**g**) Plastid with crystalloids and large plastoglobuli, mitochondria, dictyosome, and plasmalemma invaginations; (**h**) Part of the cell with a chromoplast with crystalloids and plastoglobuli, mitochondria, many vesicles, and plasmalemma invaginations; va—vacuole; p—plastid; m—mitochondrion; cw—cell wall; d—dictyosome; v—vesicle; is—intercelluar space; cr—crystalloid; g—plastoglobule. Scale bars: 5 µm (**a**,**b**), 1 µm (**c**–**e**), 0.5 µm (**f**–**h**).

In the anthesis stage, the thin-walled epidermal cells had large central and numerous smaller vacuoles, electron-dense cytoplasm, and numerous mitochondria (Figure 7a).

The cells of the nectar-producing parenchyma also had thin cell walls, different-sized vacuoles, numerous different-shaped plastids, abundant mitochondria with a transparent matrix with a few or many cristae (Figure 7b,c), numerous ribosomes, and smooth endoplasmic reticulum (ER) located near the cell walls (Figure 7d). Different-sized spaces were visible between adjacent cells of the nectariferous parenchyma (Figure 7a,b). Peripheral reticulum was observed in pleomorphic plastids (Figure 7d). The plastids in some cells formed clusters and were interconnected (Figure 7e). Many plastids had the characteristics of globular chromoplasts and contained different-sized plastoglobuli (Figure 7d–f). Crystalline chromoplasts with visible elongated crystalloids represented another type of plastids (Figure 7g). Occasionally, the crystalloids were connected with plastoglobuli. Only a few plastids contained starch grains in the nectar secretion stage (Figure 7h). Mitochondria were often located in close proximity to the chromoplasts (Figure 7c–h). Dictyosomes were observed in the peripheral cytoplasm (Figure 7g). Multiple invaginations were visible in the plasmalemma (Figure 7g). In its vicinity, there were different-sized vesicles fused with this outer cytoplasmic membrane (Figure 7h). Plasmodesmata were present in the walls of adjacent nectariferous parenchyma cells (Figure 7d,h).

## 4. Discussion

*Dracocephalum moldavica* flowers offer pollinators such visual attractants as the shape and colour of the corolla and an intense scent emitted by trichomes and papillae as well as food attractants, i.e., nectar and pollen.

The corolla is characterised by different coloured zones: the upper lip and the lower lip margins are intensely blue or purple, the central part of the lower lip is white, and the spots on this lip are intensely purple. As shown by Reverté et al. [34], the colour purple is preferred by bees in addition to pink and UV-yellow. Other researchers have found that bees have trichromate vision and are sensitive to ultraviolet, blue, and green [35,36,37,38]. These three predominant colours can be found in *D. moldavica* flowers. They are visible in various parts of the flowers. The central part usually has small colourful spots or lines standing out from the lighter background and serving as floral guides, helping insects to find the flowers and food sources [39,40]. Other authors emphasise that the colour contrasts within a flower and the flower contrast against its background are very important signals for bees [41,42]. These traits can be found in *D. moldavica* flowers, which are mainly pollinated by bees.

The epidermis of *D. moldavica* petals comprised different-sized conical cells (papillae). The longest papillae emitting essential oil were stained with anthocyanin and were observed within the coloured spots on the lower lip. As shown in literature reports, conical cells have an impact on the attractiveness of the flower to visiting insects, which increases pollination success. Papillae can perform various functions in flowers. They influence the petal colour, pollinator’s grip on the flower surface, petal wettability, petal reflexing by the formation of a velvety surface, and floral scent production [43,44].

Glandular trichomes present on various corolla parts, the ovary, and the nectary are responsible for the production of the intense scent of *D. moldavica* flowers. In previous studies, we described numerous essential oil-secreting peltate and capitate trichomes present on the abaxial surface of the lower lip, calyx, bracts, leaves, and stems of *D. moldavica* [45]. The capitate trichomes detected on the surface of nectaries had a similar structure to that of the long capitate trichomes present on the *D. moldavica* corolla and leaves.

Annular floral nectaries in some Lamiaceae representatives are characterised by the presence of a longer ventral lobe, whose abaxial surface is the main site of modified stomata. This nectary structure is characteristic of the *D. moldavica* flowers analysed in the present study. Previously, a similar nectary gland structure was observed in, e.g., some *Salvia* species [16,46], *Rosmarinus officinalis* [16], *Melissa officinalis* [17], *Hyssopus officinalis*, and *Nepeta foliosa* [15].

The number of nectarostomata in the *D. moldavica* nectary was in the range of 20–33, which was slightly higher than the values reported for some other species from this family (10–30) by Petanidou et al. [14] and Zhang et al. [16]. The number of nectarostomata in Lamiaceae plants shown by various authors was found to vary widely in the range of 4–90 [14,15,16,17,20]. Interestingly, the number of modified stomata in *Salvia triloba* and *S. verbenaca* nectaries was shown to be >90 [14]. In the present study, we observed the non-synchronous functioning of the *D. moldavica* nectarostomata. Previously, many authors also reported asynchrony in stomatal development in various plant species [12,16,47,48].

The floral nectary in *D. moldavica* is supplied by the phloem and xylem. Floral nectaries in representatives of the family Lamiaceae can be supplied by both xylem and phloem, phloem alone, or none at all [13,18,19,46,49,50]. Studies of the nectaries of many Lamiaceae species demonstrated the presence of the phloem and xylem in vascular bundles in only approximately 15% of plants [13,16,50,51]. In several *Salvia* species, which are representatives of the same subfamily Mentheae as *D. moldavica*, the phloem and xylem were found to be present in the nectary innervation [13,16,49]. In contrast, only phloem was observed in floral nectaries of other *Salvia* species, *Origanum vulgare*, and *R. officinalis* (the Mentheae subfamily as well) [13,19,50,52], whereas the nectariferous tissue in *Mentha haplocalyx* had no special vascular bundles [53]. These results show that the nectary vasculature in the Mentheae subfamily is not largely connected with their phylogenetic affinities.

Capitate glandular trichomes were present on the surface of the *D. moldavica* nectary. Similarly, capitate or peltate trichomes on the nectary glands of other Lamiaceae species were observed by various authors [16,21,22,23]. As suggested by the researchers, nectary trichomes may be involved in the production of volatile substances and can be an important diagnostic trait in the taxonomy and systematics of Lamiaceae family representatives.

The analysis of the ultrastructure of the *D. moldavica* nectary cells during the flowering stage revealed many cellular features that were described previously by various researchers in studies of nectaries of other Lamiaceae plants. The nectary epidermal cells of the species analysed in the present study contained a large central vacuole, as in the case of *R. officinalis* [19] and *Salvia farinacea* [16] described previously.

The nectariferous parenchyma of the *D. moldavica* cells had dense cytoplasm, numerous vacuoles, mitochondria, plastids, ribosomes, ER, and thin cell walls with plasmodesmata. The pleomorphic and often electron-opaque plastids formed clusters, as in the case of the nectaries of *R. officinalis* [19] and *S. farinacea* [16]. Starch grains were present in the analysed *D. moldavica* nectary tissues only in the flower buds, which corresponded with the bright yellow colour of this gland. As a rule, no starch was observed in the nectary cells in this species during the nectar secretion phase. The presence of starch in the nectaries of other Lamiaceae only in the pre-secretory phase and its decomposition during nectar secretion were also reported by Xin et al. [54], Teng and Hu [55], and Zhang et al. [16]. In turn, there were certain amounts of starch in *R. officinalis* plastids in the nectar secretion stage and after the end of nectar secretion [19]. In the nectar secretion stage, there were globular and crystalline carotenoid-containing chromoplasts in the nectary-producing parenchyma cells of *D. moldavica*, which was associated with the more intense yellow pigmentation of the nectaries.

The plasmalemma of the nectary cells in the analysed plants exhibited large invaginations and numerous vesicles in their vicinity. Plasmalemma invaginations in nectariferous parenchyma cells were also observed in *R. officinalis* by Zer and Fahn [19].

The nectariferous parenchyma cells of *D. moldavica* exhibited the presence of ER, dictyosomes, and numerous vesicles fused with the plasmalemma, which is indicative of granulocrine secretion. The presence of plasmodesmata in cell walls evidences the symplastic transport of sugars [56].

The histochemical and fluorescence assays showed the presence of lipid compounds (total lipids, acidic, and neutral lipids) and phenolic compounds (total phenols, phenolic acids, and flavonoids) in the tissues and glandular trichomes of the *D. moldavica* nectary. Various authors also reported the presence of similar groups of metabolites in nectaries of other plant species [57,58,59,60]. Similar to the present findings, these researchers showed that the metabolites were located in the nectary epidermis and/or parenchyma; next, the compounds penetrated into the secreted nectar. The literature data indicate that essential oils and phenolic compounds can act as repellents deterring herbivores, parasites, and nectar robbers [61,62,63] and have antifungal and antibacterial properties [64,65]. Astringent and toxic phenolic substances may be associated with pollinator attraction through the intensification of scent perception [66]; they may also be involved in ultraviolet light protection through the absorption of radiation, reducing damage by acting as a sunscreen [67]. As reported by dos Santos Silva et al. [68], the phenolic compounds present in nectary tissues may play a role in the oxidation of the nectary region, which ultimately limits the growth and passage of the pollen tube and prevents ovule fertilisation in such partenocarpic plants as the *Musa* spp. Additionally, some phenolic compounds, e.g., flavonoids, which are common in the nectary and nectar, were shown to be preferred by honeybees in preference tests and upregulate detoxification and immunity genes [64,69].

Lipids, which were identified in the *D. moldavica* epidermis, parenchyma, and trichomes, are important metabolites increasing the caloric content of nectar. As suggested by Kram et al. [70], the presence of lipids in nectary cells contributes to the content of more energy-efficient lipids in the nectar produced in the nectary and is, therefore, more attractive to bees. We confirmed the presence of acidic and neutral lipids, including essential oils, in the *D. moldavica* nectary tissues. As reported by Desbois and Smith [71], acidic lipids, as free fatty acids, exhibit antibacterial activity consisting of the disruption of bacterial membrane structures. A similar function is also attributed to terpenoids, which are components of essential oils [72,73]. The essential oil present in the *D. moldavica* nectary epidermis presumably acts as an odour attractant for insect pollinators. This role of essential oils was also reported by Raguso [74,75] and Dodoš et al. [76]. Moreover, since the sensitivity to secondary metabolites differs between floral visitors, unpalatable and unattractive compounds may function as taste filters eliminating ineffective pollinators and nectar robbers [65]. The presence of secondary metabolites in the nectary and nectar provides a direct health benefit not only to plants but also to their pollinators. Biller et al. [77] and Richardson et al. [78] claim that the consumption of these metabolites by bees reduces the pathogen load and increases the survivorship in these bees. In turn, Gherman et al. [79] suggest that some pollinators may intentionally visit selected flowers to self-medicate, thus reducing infection and increasing health. As in the case of the *D. moldavica*, the cells of the nectaries of other taxa most often contain a combination of bioactive compounds with synergistic or antagonistic activity, depending on the final compound formed and the concentration of the individual components [80]. As shown by Köhler et al. [81], the effect of deterrence depends on concentrations of both sugar and toxic compounds: pollinators are more tolerant to toxic compounds at a higher concentration of sugars in the nectar. The manipulation of pollinator behaviour may improve the reproductive success of plants, and protection against diseases may help the fitness of pollinators [65].

Similarly, as reported in the present work, earlier studies conducted by various authors demonstrated mixed secretion by nectaries in other plant species [82]. It has been shown that floral nectaries produced sugars, lipids, and phenolic compounds in some Anacardiaceae [83], Bignoniaceae [82], Celastraceae [84], Fabaceae [85], and Rosaceae [86] plants.

## 5. Conclusions

We have shown that essential oil, which determines the intense scent of *D. moldavica*, is emitted not only by all vegetative parts of the shoot [4,45] but also by petals (with glandular trichomes and papillae) and floral nectaries. Our study provides the first description of the production of nectar and essential oil by the nectaries in the family Lamiaceae. Volatile substances produced by flowers and nectaries, together with the presence of the nectar reward, have a positive impact on the attractiveness of the flowers of this species of insect pollinators, which is reflected in the pollination and reproductive success of this species. The essential oil and phenolic compounds present in the nectary tissue may also provide protection against herbivores and pathogens. They can also act as repellents against undesirable insect pollinators. *D. moldavica* nectaries are embedded around the ovary base, which is typical of the Lamiaceae family members. The nectaries of this species, which are characterised by pre-secretory starch accumulation, release nectar through modified stomata. Nectar secretion and transport proceed via the granulocrine and symplastic pathways, respectively. These results may improve the knowledge and understanding of plant–pollinator interactions. Furthermore, analyses of *D. moldavica* nectaries may be important for systematics and evolutionary studies of the Lamiaceae family.

## Figures and Tables

**Table 1:** Measurements of the *Dracophalum moldavica* nectary.

Feature	Flower Stage					
	Bud			Anthesis		
	Range	Average	SD	Range	Average	SD
Height of the longer nectary lobe (µm)	950.4–1077.1	995.8	±56.1	1198.6–1262.3	1220.5	±36.2
Longer diameter of the nectary (µm)	920.5–1086.8	978.7	±93.7	1173.3–1187.9	1181.9	±7.7
Shorter diameter of the nectary (µm)	865.4–871.6	868.9	±3.2	906.0–960.2	939.6	±29.4

## Data Availability

The data presented in this study are available on request from the corresponding author.

## References

[1] Christenhusz M.J.M., Fay M.F., Chase M.W. (2017). Plants of the World. An Illustrated Encyclopedia of Vascular Plants.

[2] Nikolova M., Traykova B., Yankova-Tsvetkova E., Stefanova T., Dzhurmanski A., Aneva I., Berkov S. (2021). Herbicide Potential of Selected Essential Oils From Plants of Lamiaceae and Asteraceae Families. Acta Agrobot..

[3] Kakasy A.Z., Lemberkovics E., Kursinszki L., Janicsak G., Szِoke E. (2002). Data to the phytochemical evaluation of Moldavian dragonhead (*Dracocephalum moldavica* L., Lamiaceae). Herba Pol..

[4] Dmitruk M., Sulborska A., Żuraw B., Stawiarz E., Weryszko-Chmielewska E. (2019). Sites of secretion of bioactive compounds in leaves of *Dracocephalum moldavica* L.: Anatomical, histochemical, and essential oil study. Braz. J. Bot..

[5] Wolski T., Kwiatkowski S., Gliński Z. (2004). Pszczelnik mołdawski (*Dracocephalum moldavica* L.)—Roślina miododajna i lecznicza. Ann. UMCS Sec. DD.

[6] Ehsani A., Mahjani M.G., Hosseini M., Safari R., Moshrefi R., Mohammad Shiri H. (2017). Evaluation of *Thymus vulgaris* plant extract as an eco-friendly corrosion inhibitor for stainless steel 304 in acidic solution by means of electrochemical impedance spectroscopy, electrochemical noise analysis and density functional theory. J. Colloid Interface Sci..

[7] Lipiński M. (2010). Pożytki Pszczele. Zapylanie I Miododajność Roślin.

[8] Dmitruk M., Weryszko-Chmielewska E., Sulborska A. (2018). Flowering and Nectar Secretion in Two Forms of the Moldavian Dragonhead (*Dracocephalum moldavica* L.)—A Plant with Extraordinary Apicultural Potential. J. Apic. Sci..

[9] Sulborska A. (2019). Rośliny Pożytkowe.

[10] Naie M., Trotus E., Lupu C., Popa D. (2016). Data and knowledge on the importance of *Dracocephalum moldavica* L. species (dragon’s head) to introduce and develop the cultivation technology. An. Stiintifice Ale Univ. Alexandru Ioan Cuza Din Iasi. Sect. II A Biol. Veg..

[11] Petanidou T., Nicolson S.W., Nepi M., Pacini. E. (2007). Ecological and Evolutionary Aspects of Floral Nectars in Mediterranean Habitats. Nectaries and Nectar.

[12] Nepi M., Nicolson S.W., Nepi M., Pacini E. (2007). Nectary Structure and Ultrastructure. Nectaries and Nectar.

[13] Dafni H., Lensky Y., Fahn A. (1988). Flower and nectar characteristics of nine species of Labiatae and their influence on honeybee visits. J. Apic. Res..

[14] Petanidou T., Goethals V., Smets E. (2000). Nectary structure of Labiatae in relation to their nectar secretion and characteristics in a Mediterranean shrub community—Does flowering time matter?. Plant Syst. Evol..

[15] Weryszko-Chmielewska E. (2000). Ecological features of flowers including nectary structure of chosen species from Lamiaceae family. Pszczeln. Zesz. Nauk..

[16] Zhang X., Sawhney V.K., Davis A.R. (2014). Annular floral nectary with oil-producing trichomes in *Salvia farinacea* (Lamiaceae): Anatomy, histochemistry, ultrastructure, and significance. Am. J. Bot..

[17] Chwil M. (2009). Flowering biology and nectary structure of *Melissa officinalis* L.. Acta Agrobot..

[18] Mačukanović-Jocić M.P., Rančić D.V., Dajić Stevanović Z.P. (2007). Floral nectaries of basil (*Ocimum basilicum*): Morphology, anatomy and possible mode of secretion. S. Afr. J. Bot..

[19] Zer H., Fahn A. (1992). Floral Nectaries of *Rosmarinus officinalis* L. Structure, Ultrastructure and Nectar Secretion. Ann. Bot..

[20] Chwil M. (2007). Flowering pattern, the structure of nectary surfaceand nectar secretion in two varieties of *Ocimum basilicum* L.. Acta Agrobot..

[21] Kartashova N.N. (1960). Selected data on the morphology of the flowers of family Labiatae. Bot. J. CCCP Acad. Sci..

[22] Naghiloo S., Gohari G.R., Nikzat Siahkolaee S., Dadpour M.R. (2015). Floral development in *Scutellaria pinnatifida* (Lamiaceae): The ontogenetic basis for sepal reduction. Plant Biol..

[23] Naghiloo S., Khodaverdi M., Nikzat Siahkolaee S., Dadpour M.R. (2014). Comparative floral development in Lamioideae (Lamiaceae): *Marrubium*, *Phlomis*, and *Stachys*. Plant Syst. Evol..

[24] O’Brien T.P., McCully M.E. (1981). The Study of Plant Structure: Principles and Selected Methods.

[25] Pearse A.G.E. (1985). Histochemistry: Theorical and Applied.

[26] Brundrett M.C., Kendrick B., Peterson C.A. (1991). Efficient lipid staining in plant material with Sudan Red 7B or Fluoral Yellow 088 in polyethylene glycol-glycerol. Biotech. Histochem..

[27] Cain A.J. (1947). The use of Nile blue in the examination of lipids. Q.J. Microsc. Sci..

[28] Jensen W.A. (1962). Botanical Histochemistry Principles and Practice.

[29] Johansen D.A. (1940). Plant microtechnique.

[30] Mabry T.J., Markham K.R., Thomas M.B. (1970). The Systematic Identification of Flavonoids.

[31] Talamond P., Verdeil J.L., Conéjéro G. (2015). Secondary metabolite localization by autofluorescence in living plant cells. Molecules.

[32] Charrière-Ladreix Y. (1976). Répartition intracellulaire du secrétat flavonique de *Populus nigra* L.. Planta.

[33] Reynolds E.S. (1963). The use of lead citrate at high pH as an electronopaque stain in electron microscopy. J. Cell Biol..

[34] Reverté S., Retana J., Gómez J.M., Bosch J. (2016). Pollinators show flower colour preferences but flowers with similar colours do not attract similar pollinators. Ann Bot..

[35] Menzel R., Backhaus. W., Gouras P. (1991). Colour Vision in Insects. Vision and Visual Dysfunction.

[36] Vorobyev M., Osorio D., Bennett A.T.D., Marshall N.J., Cuthill I.C. (1998). Tetrachromacy, oil droplets and bird plumage colours. J. Comp. Physiol. A.

[37] Dyer A.G., Boyd-Gerny S., Shrestha M., Lunau K., Garcia J.E., Koethe S., Wong B.B.M. (2016). Innate colour preferences of the Australian native stingless bee *Tetragonula carbonaria Sm*. J. Comp. Physiol. A.

[38] Papiorek S., Junker R.R., Alves-dos-Santos I., Melo G.A.R., Amaral-Neto L.P., Sazima M., Wolowski M., Freitas L., Lunau K. (2016). Bees, birds and yellow flowers: Pollinator-dependent convergent evolution of UV patterns. Plant Biol..

[39] de Ibarra N.H., Langridge K.V., Vorobyev M. (2015). More than colour attraction: Behavioural functions of flower patterns. Curr. Opin. Insect Sci..

[40] Lunau K., Wester P. (2017). Mimicry and deception in pollination. Adv. Bot. Res..

[41] Schmidt V., Martin Schaefer H., Winkler H. (2004). Conspicuousness, not colour as foraging cue in plant–animal signalling. Oikos.

[42] van der Kooi C.J., Dyer A.G., Kevan P.G., Lunau K. (2018). Functional significance of the optical properties of flowers for visual signaling. Ann. Bot..

[43] Whitney H.M., Bennett K.M., Dorling M., Sandbach L., Prince D., Chittka L., Glover B.J. (2011). Why do so many petals have conical epidermal cells?. Ann. Bot..

[44] Whitney H.M., Poetes R., Steiner U., Chittka L., Glover B.J. (2011). Determining the contribution of epidermal cell shape to petal wettability using isogenic *Antirrhinum* lines. PLoS ONE.

[45] Dmitruk M., Weryszko-Chmielewska E. (2010). Morphological differentiation and distribution of non-glandular and glandular trichomes on *Dracocephalum moldavicum* L. shoots. Acta Agrobot..

[46] Kumari D.S. (1986). Evolution of floral nectary in Lamiaceae. P. Indian AS-Plant Sci..

[47] Davis A.R., Gunning B.E.S. (1992). The modified stomata of the floral nectary of *Vicia faba* L. 1. Development, anatomy and ultra-structure. Protoplasma.

[48] Gaffal K.P., Friedrichs G.J., El-Gammal S. (2007). Ultrastructural evidence for a dual function of the phloem and programmed cell death in the floral nectary of *Digitalis purpurea*. Ann. Bot..

[49] Kartashova N.N. (1965). Stroeniei Funktsiya Nektarnikov Tsvetka Dvodol’nykh Rastenii.

[50] Frei E. (1955). Die Innervierung der floralen Nektarien dikotyler Pflanzenfamilien. Ber. Schweiz. Bot. Ges..

[51] Rudall P. (1981). Flower anatomy of subtribe Hyptidinae (Labiatae). Bot. J. Linn. Soc..

[52] Yanbin D., Hong W., Yong L. (1997). Developmental and anatomical studies on the floral nectaries in *Origanum vulgare* Linn. Acta Bot. Boreal. Occid. Sin..

[53] Shen-Zonggen S., Wenzhe L., Zhenghai H. (1994). Developmental and anatomic studies on the floral nectaries of *Mentha haplocalyx*. Acta Bot. Boreal. Occid. Sin..

[54] Xin H., Chu Q.G., Hu Z.H. (2000). Anatomical studies on the development of the floral nectary in *Thymus quinquecostatus* Celak. J. Plant Resour. Environ..

[55] Teng H.M., Hu Z.H. (2003). Developmental and anatomical studies on the floral nectaries in *Perilla frutescens*. Xibei Zhiwu Xuebao.

[56] Tölke E.D., Capelli N.D.V., Pastori T., Alencar A.C., Cole T.C., Demarco D., Mérillon J.-M., Ramawat K.G. (2020). Diversity of Floral Glands and Their Secretions in Pollinator Attraction. Co-Evolution of Secondary Metabolites.

[57] Konarska A. (2020). Microstructure of floral nectaries in *Robinia viscosa* var. hartwigii (Papilionoideae, Fabaceae)—A valuable but little-known melliferous plant. Protoplasma.

[58] Konarska A. (2022). Morphological, anatomical, ultrastructural, and histochemical study of flowers and nectaries of *Iris sibirica* L.. Micron.

[59] Chitchak N., Stewart A.B., Traiperm P. (2022). Functional Ecology of External Secretory Structures in *Rivea ornata* (Roxb.) Choisy (Convolvulaceae). Plants.

[60] Gardoni L.C.D.P., Santana R.M., Brito J.C.M., Ramos L.X., Araújo L.A., Bastos E.M.A.F., Calaça P. (2022). Content of phenolic compounds in monofloral aroeira honey and in floral nectary tissue. Pesqui. Agropecu. Bras..

[61] Stevenson P.C., Nicolson S.W., Wright G.A. (2017). Plant secondary metabolites in nectar: Impacts on pollinators and ecological functions. Funct. Ecol..

[62] Ebadollahi A., Ziaee M., Palla F. (2020). Essential oils extracted from different species of the Lamiaceae plant family as prospective bioagents against several detrimental pests. Molecules.

[63] War A.R., Buhroo A.A., Hussain B., Ahmad T., Nair R.M., Sharma H.C., Mérillon J.-M., Ramawat K.G. (2020). Plant Defense and Insect Adaptation with Reference to Secondary Metabolites. Co-Evolution of Secondary Metabolites.

[64] Palmer-Young E.C., Farrell I.W., Adler L.S., Milano N.J., Egan P.A., Junker R.R., Irwin R.E., Stevenson P.C. (2019). Chemistry of floral rewards: Intra- and interspecific variability of nectar and pollen secondary metabolites across taxa. Ecol. Monogr..

[65] Nicolson S.W. (2022). Sweet solutions: Nectar chemistry and quality. Philos. T. R. Soc. B.

[66] Kowalkowska A.K., Pawłowicz M., Guzanek P., Krawczyńska A.T. (2018). Floral nectary and osmophore of *Epipactis helleborine* (L.) Crantz (Orchidaceae). Protoplasma.

[67] Kumar S., Abedin M., Singh A.K., Das S., Lone R., Shuab R., Kamili A.N. (2020). Role of Phenolic Compounds in Plant-Defensive Mechanisms. Plant Phenolics in Sustainable Agriculture Singapore.

[68] dos Santos Silva M., Santana A.N., dos Santos-Serejo J.A., Ferreira C.F., Amorim E.P. (2022). Morphoanatomy and Histochemistry of Septal Nectaries Related to Female Fertility in Banana Plants of the ‘Cavendish’ Subgroup. Plants.

[69] Liao L.H., Wu W.-Y., Berenbaum M.R. (2017). Behavioral responses of honey bees (*Apis mellifera*) to natural and synthetic xenobiotics in food. Sci. Rep..

[70] Kram B.W., Bainbridge E.A., Perera M.A.D.N., Carter C. (2008). Identification, cloning and characterization of a GDSL lipase secreted into the nectar of *Jacaranda mimosifolia*. Plant Mol. Biol..

[71] Desbois A.P., Smith V.J. (2010). Antibacterial free fatty acids: Activities, mechanisms of action and biotechnological potential. Appl. Microbiol. Biotechnol..

[72] Wang C.Y., Chen Y.W., Hou C.Y. (2019). Antioxidant and antibacterial activity of seven predominant terpenoids. Int. J. Food Prop..

[73] Yamaguchi T. (2022). Antibacterial effect of the combination of terpenoids. Arch. Microbiol..

[74] Raguso R.A. (2004). Why are some floral nectars scented?. Ecology.

[75] Raguso R.A., Baser K.H.C., Buchbauer G. (2020). Functions of Essential Oils and Natural Volatiles in Plant-Insect Interactions. Handbook of Essential Oils: Science, Technology, and Applications.

[76] Dodoš T., Janković S., Marin P.D., Rajčević N. (2021). Essential Oil Composition and Micromorphological Traits of *Satureja montana* L., *S. subspicata* Bartel ex Vis., and *S. kitaibelii* Wierzb. Ex Heuff. Plant Organs. Plants.

[77] Biller O.M., Adler L.S., Irwin R.E., McAllister C., Palmer-Young E.C. (2015). Possible synergistic effects of thymol and nicotine against *Crithidia bombi* parasitism in bumble bees. PLoS ONE.

[78] Richardson L.L., Adler L.S., Leonard A.S., Andicoechea J., Regan K.H., Anthony W.E., Manson J.S., Irwin R.E. (2015). Secondary metabolites in floral nectar reduce parasite infections in bumblebees. Proc. R. Soc. Lond. B Biol. Sci..

[79] Gherman B.I., Denner A., Bobis O., Dezmirean D.S., Marghitas L.A., Schluns H., Moritz R.F.A., Erler S. (2014). Pathogen-associated self-medication behavior in the honeybee *Apis mellifera*. Behav. Ecol. Sociobiol..

[80] Roy R., Schmitt A.J., Thomas J.B., Carter C.J. (2017). Nectar biology: From molecules to ecosystems. Plant Sci..

[81] Köhler A., Pirk C.W.W., Nicolson S.W. (2012). Honeybees and nectar nicotine: Deterrence and reduced survival versus potential health benefits. J. Insect. Physiol..

[82] Machado S.R., Souza C.V., Guimarães E. (2017). A reduced, yet functional, nectary disk integrates a complex system of floral nectar secretion in the genus *Zeyheria* (Bignoniaceae). Acta Bot. Bras..

[83] Tölke E.D., Bachelier J.B., Lima E.A., Galetto L., Demarco D., Carmello-Guerreiro S.M. (2018). Diversity of floral nectary secretions and structure, and implications for their evolution in Anacardiaceae. Bot. J. Linn. Soc..

[84] Mercandante-Simões M.O., Paiva E.A.S. (2016). Anatomy and ultrastructure of the floral nectary of *Tontelea micrantha* (Celastraceae: Salacioideae): Floral nectary of *Tontelea micrantha*. Plant Spec. Biol..

[85] Paiva E., Machado S.R. (2008). The floral nectary of *Hymenaea stigonocarpa* (Fabaceae, Caesalpinioideae): Structural aspects during floral development. Ann. Bot..

[86] Weryszko-Chmielewska E., Sulborska-Różycka A., Sawidis T. (2022). Structure of the nectary in *Chaenomeles japonica* (Thunb.) Lindl. Ex Spach. in different stages of flowering with focus on nectar secretion. Protoplasma.

